# A systematic review of the development and application of home cage monitoring in laboratory mice and rats

**DOI:** 10.1186/s12915-023-01751-7

**Published:** 2023-11-13

**Authors:** Pia Kahnau, Paul Mieske, Jenny Wilzopolski, Otto Kalliokoski, Silvia Mandillo, Sabine M. Hölter, Vootele Voikar, Adriana Amfim, Sylvia Badurek, Aleksandra Bartelik, Angela Caruso, Maša Čater, Elodie Ey, Elisabetta Golini, Anne Jaap, Dragan Hrncic, Anna Kiryk, Benjamin Lang, Natasa Loncarevic-Vasiljkovic, Hamid Meziane, Aurelija Radzevičienė, Marion Rivalan, Maria Luisa Scattoni, Nicolas Torquet, Julijana Trifkovic, Brun Ulfhake, Christa Thöne-Reineke, Kai Diederich, Lars Lewejohann, Katharina Hohlbaum

**Affiliations:** 1 German Federal Institute for Risk Assessment (BfR), German Centre for the Protection of Laboratory Animals (Bf3R), Berlin, Germany; 2 Institute of Animal Welfare, Animal Behavior and Laboratory Animal Science, Department of Veterinary Medicine, Freie Universität Berlin, Berlin, Germany; 3 Science of Intelligence, Research Cluster of Excellence, Marchstr. 23, 10587 Berlin, Germany; 4 Department of Experimental Medicine, University of Copenhagen, Copenhagen, Denmark; 5 Institute of Biochemistry and Cell Biology, National Research Council CNR, Rome, Italy; 6 Helmholtz Zentrum München, German Research Centre for Environmental Health, Munich, Germany; 7 Neuroscience Center, Helsinki Institute of Life Science (HiLIFE), University of Helsinki, Helsinki, Finland; 8 Faculty of Veterinary Medicine, Spiru Haret University, Bucharest, Romania; 9 Preclinical Phenotyping Facility, Vienna Biocenter Core Facilities (VBCF), member of the Vienna Biocenter (VBC), Vienna, Austria; 10 International Clinical Research Center, St. Anne’s University Hospital Brno, Brno, Czech Republic; 11 Istituto Superiore Di Sanità, Research Coordination and Support Service, Rome, Italy; 12 Department of Animal Science, Biotechnical Faculty, University of Ljubljana, Ljubljana, Slovenia; 13 Université de Strasbourg, CNRS, Inserm, Institut de Génétique et de Biologie Moléculaire et Cellulaire UMR 7104- UMR-S 1258, Illkirch, 67400 France; 14 Institute of Medical Physiology “Richard Burian”, Faculty of Medicine, University of Belgrade, Belgrade, Serbia; 15 Laboratory of Preclinical Testing of Higher Standard, Neurobiology Center, Nencki Institute of Experimental Biology, Polish Academy of Science, Warsaw, Poland; 16 iNOVA4Health, NOVA Medical School, Faculdade de Ciências Médicas, NMS, FCM, Universidade Nova de Lisboa, Lisbon, Portugal; 17 Université de Strasbourg, CNRS, INSERM, Institut Clinique de La Souris (ICS), CELPHEDIA, PHENOMIN, 1 Rue Laurent Fries, Illkirch, 67404 France; 18 Lithuanian University of Health Sciences, Medical Academy, Institute of Physiology and Pharmacology, Kaunas, Lithuania; 19 Research Institute for Experimental Medicine (FEM) and NeuroCure Cluster of Excellence, Animal Behaviour Phenotyping Facility, Charité - Universitätsmedizin Berlin, Berlin, Germany; 20 Université de Strasbourg, CNRS, Inserm, IGBMC, Institut Clinique de la Souris (ICS), CELPHEDIA, PHENOMIN, UMR 7104- UMR-S 1258, Illkirch, 67400 France; 21 Department of Veterinary Medicine, Faculty of Agriculture, University of East Sarajevo, East Sarajevo, Bosnia and Herzegovina; 22 Div. Clinical Physiology, Department of Laboratory Medicine, Karolinska Institutet, Stockholm, Sweden

**Keywords:** Home cage monitoring, Rodents, Rats, Mice, Sex bias, Behavior, Physiology, Refinement, Animal welfare, History

## Abstract

**Background:**

Traditionally, in biomedical animal research, laboratory rodents are individually examined in test apparatuses outside of their home cages at selected time points. However, the outcome of such tests can be influenced by various factors and valuable information may be missed when the animals are only monitored for short periods. These issues can be overcome by longitudinally monitoring mice and rats in their home cages. To shed light on the development of home cage monitoring (HCM) and the current state-of-the-art, a systematic review was carried out on 521 publications retrieved through PubMed and Web of Science.

**Results:**

Both the absolute (~ × 26) and relative (~ × 7) number of HCM-related publications increased from 1974 to 2020. There was a clear bias towards males and individually housed animals, but during the past decade (2011–2020), an increasing number of studies used both sexes and group housing. In most studies, animals were kept for short (up to 4 weeks) time periods in the HCM systems; intermediate time periods (4–12 weeks) increased in frequency in the years between 2011 and 2020. Before the 2000s, HCM techniques were predominantly applied for less than 12 h, while 24-h measurements have been more frequent since the 2000s. The systematic review demonstrated that manual monitoring is decreasing in relation to automatic techniques but still relevant. Until (and including) the 1990s, most techniques were applied manually but have been progressively replaced by automation since the 2000s. Independent of the year of publication, the main behavioral parameters measured were locomotor activity, feeding, and social behaviors; the main physiological parameters were heart rate and electrocardiography. External appearance-related parameters were rarely examined in the home cages. Due to technological progress and application of artificial intelligence, more refined and detailed behavioral parameters have been investigated in the home cage more recently.

**Conclusions:**

Over the period covered in this study, techniques for HCM of mice and rats have improved considerably. This development is ongoing and further progress as well as validation of HCM systems will extend the applications to allow for continuous, longitudinal, non-invasive monitoring of an increasing range of parameters in group-housed small rodents in their home cages.

**Supplementary Information:**

The online version contains supplementary material available at 10.1186/s12915-023-01751-7.

## Background

In biomedical research, laboratory rodents traditionally are removed from their home cages for defined periods of time (from a few minutes up to several hours) and placed in experimental apparatuses to measure parameters of interest. The experimental apparatuses usually are unfamiliar to the animals and, therefore, represent novel environments. In several cases, experimental designs include familiarization sessions to reduce the potential impact of stress and anxiety before collecting the parameters of interest. Common examples for monitoring behavioral parameters, such as anxiety-related behavior or locomotor activity, are the Open Field and the Elevated Plus Maze tests [1]. Both tests are based on mice’s and rats’ natural avoidance of open areas (Open Field) where risk of predation is high, as well as unsafe enclosures (height with no protection in the Elevated Plus Maze), measuring exploration and locomotor activity as expressions of anxiety-like behavior [2]. However, such brief tests, performed outside of the home cage, may not adequately reflect the complexity of, for example, anxiety-like behavior [3–7]. Moreover, the behaviors displayed by an animal in these tests also depend on unrelated factors [8] and it has been shown that the reproducibility of test results obtained in this way is low [9].

The same applies to the collection of other than behavioral data outside the home cage. If, for instance, body temperature is measured using a rectal probe, an animal will generally be removed from its home cage and be hand-restrained for the duration of the measurements, which can result in a stress response and stress-associated hyperthermia [10].

When the animals are tested outside of their home cage, the time of day can play a critical role, too. Although rats and mice are nocturnal/crepuscular animals, experiments are often carried out during the daytime when lights are turned on [11–13]. In addition, it needs to be considered which behaviors are observed at what time. Mice show higher activity in an Open Field test during the dark phase when compared to the light phase [14]. Mice also consume less food during the light phase and are less motivated to work for rewards when compared to dark phase performance [15].

Other disadvantages of tests performed outside the animals’ home cage are that the animals are handled by an experimenter and separated from their familiar social group, which may negatively influence both animal welfare and data quality [16–19]. Another influencing factor is the laboratory environment. In 1999, selected strains of mice were tested under standardized conditions (same test equipment, protocols, and environmental variables) in three different laboratories. The results showed that the behavior of the mouse strains depended on the laboratory. The authors warn that experiments performed to characterize mouse strains may be laboratory dependent and therefore less strain specific [4]. To date, the reproducibility crisis remains unresolved and various approaches are discussed and pursued to improve the reliability of scientific data [4, 7, 20–24]. One approach that can be used to minimize variability between laboratories is to monitor the animals in their familiar environment (i.e., in their home cage). Krackow and colleagues were able to show that both the activity and spatial learning of mice tested in the home cage was constant across different laboratories [25].

Housing conditions for laboratory animals vary considerably between animal facilities around the world and there is no standard definition of a home cage. However, a home cage can generally be considered as the environment where the animals spend most of their lifetimes [26, 27]. In the European Union, this environment must meet the minimum requirements defined in the Directive 2010/63/EU. The Directive stipulates that social animals should be kept in groups whenever possible [28]. The minimum floor area of a home cage should be adjusted depending on the species, the number of animals, and their body weight (size). Food, water, bedding, and nesting material must be provided [28]. Furthermore, the home cage should be structured in a way that allows a wide range of natural behaviors to be exhibited [28]. However, a lack of space, social contact, environmental enrichment, and novelty aspects prevent the animals from displaying their full behavioral repertoire and can result in major animal welfare issues, as observed by the occurrence of stereotypic behaviors and increased aggression.

A broad range of parameters can be monitored in the home cage using manual or automated techniques, e.g., a variety of behavioral parameters such as activity [29, 30], social behavior [31–33], learning and memory [34–36], feeding [37–39], and physiological parameters like heart rate or body temperature [40–43]. Manual techniques used for home cage monitoring (HCM) include live observations or analyses of videos recorded by camera-based systems, which can be complemented or substituted by automated HCM techniques. Automated HCM techniques have been implemented in various commercial systems. A range of systems were described in a review by Richardson et al. [44]. For example, a combination of light-emitting diodes (LEDs), infrared light sensors, and a camera allows for analyzing, among others, physical activity or learning behavior [35, 45]. Electrical capacitance technology makes it possible to measure physical activity or rest [46, 47]. When the animals move in the cage, changes in a weak electromagnetic field can be utilized to track their movements. However, these systems do not allow the acquisition of individual data from group-housed animals. Data from individuals, also when they are kept in social groups, can be generated using radio-frequency identification (RFID) systems. For instance, the activity of 20–40 mice can be automatically measured in a large semi-naturalistic home cage [48, 49]. RFID systems enable testing learning and memory of mice and rats using operant conditioning corners that grant or deny access to water [36, 50, 51] and performing preference tests in the home cage [52, 53]. RFID systems can be combined with other techniques such as depth-sensing infrared camera for automatic individual tracking of animals [54].

HCM offers several advantages for both data quality [25, 55, 56] and animal welfare [46, 57, 58]. Ideally, group-housed animals are observed and/or tested in their familiar environment 24/7 and remain undisturbed by the experimenters. HCM has grown in importance and demands have changed over the last decades driven by technology and digitalization. However, automated 24-h measurements for long-term periods still seem to be a major challenge.

In 2021, European researchers joined forces in a project titled “Improving biomedical research by automated behaviour monitoring in the animal home-cage” (TEATIME: cost-teatime.org) in order to promote the further use and development of HCM systems. On this website, a comprehensive catalog of currently existing HCM systems is provided (cost-teatime.org/about/technologies), which will be further updated. Approximately at the same time, another initiative called “Translational Digital Biomarkers” was launched within the North American 3Rs Collaborative (www.na3rsc.org/tdb). To identify past trends in and the current state of HCM, a systematic review was carried out in close collaboration with the COST Action TEATIME network. The following research questions were addressed: How have techniques and applications for home cage monitoring of laboratory mice and rats developed over time? Has the degree of automatization for monitoring behavioral, physiological, and external appearance-related parameters changed?

## Results

### Overview of the studies

**Fig. 1:**
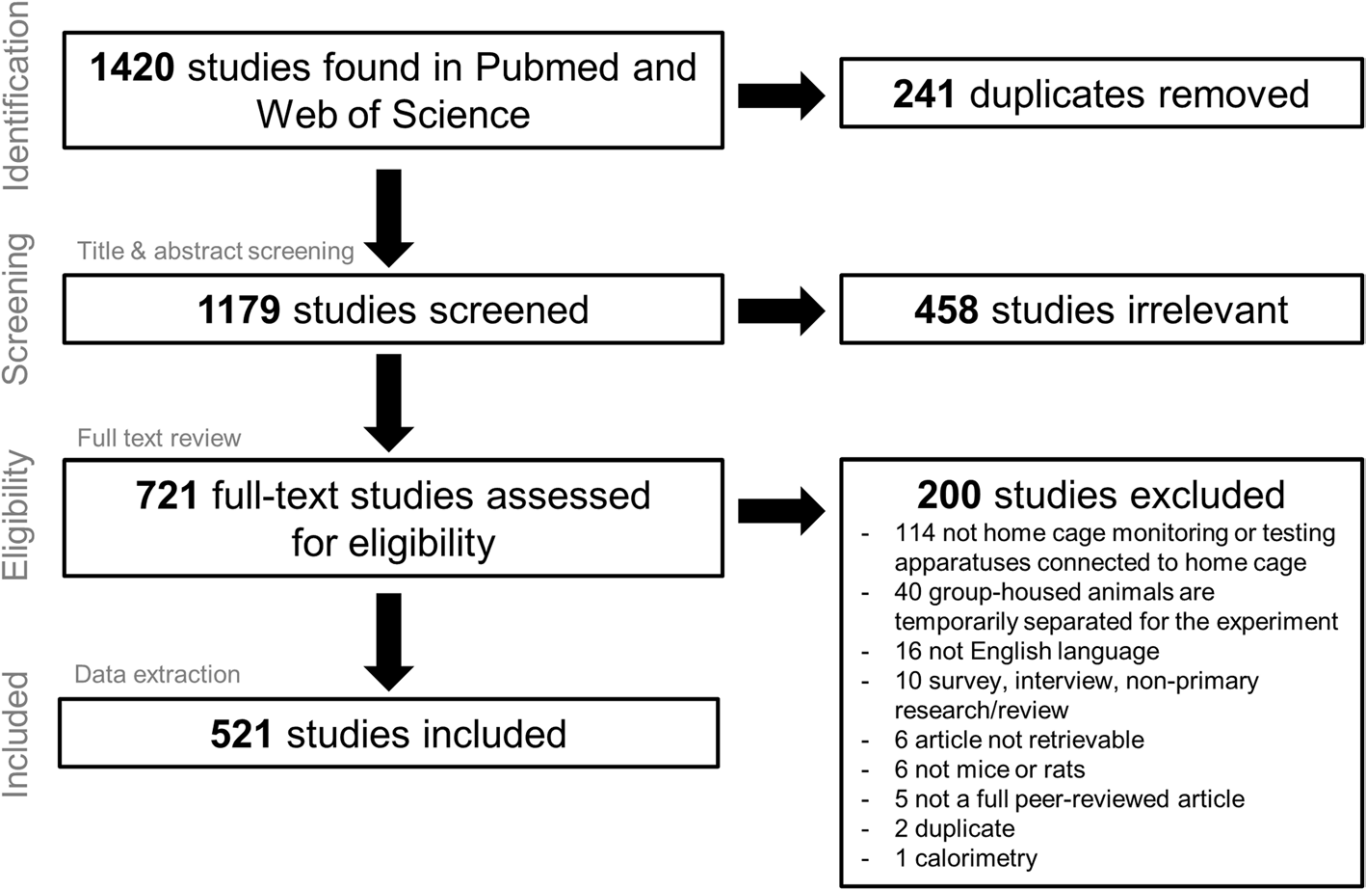
PRISMA flow diagram

The search through PubMed and Web of Science retrieved 1079 and 341 references, respectively. Figure 1 shows the PRISMA flow diagram. A total of 241 duplicates was removed. Titles and abstracts of 1179 references were screened, of which the full text for 721 references was assessed for eligibility. Five hundred twenty-one references were retained for data extraction.

### Increasing number of publications on HCM

**Fig. 2:**
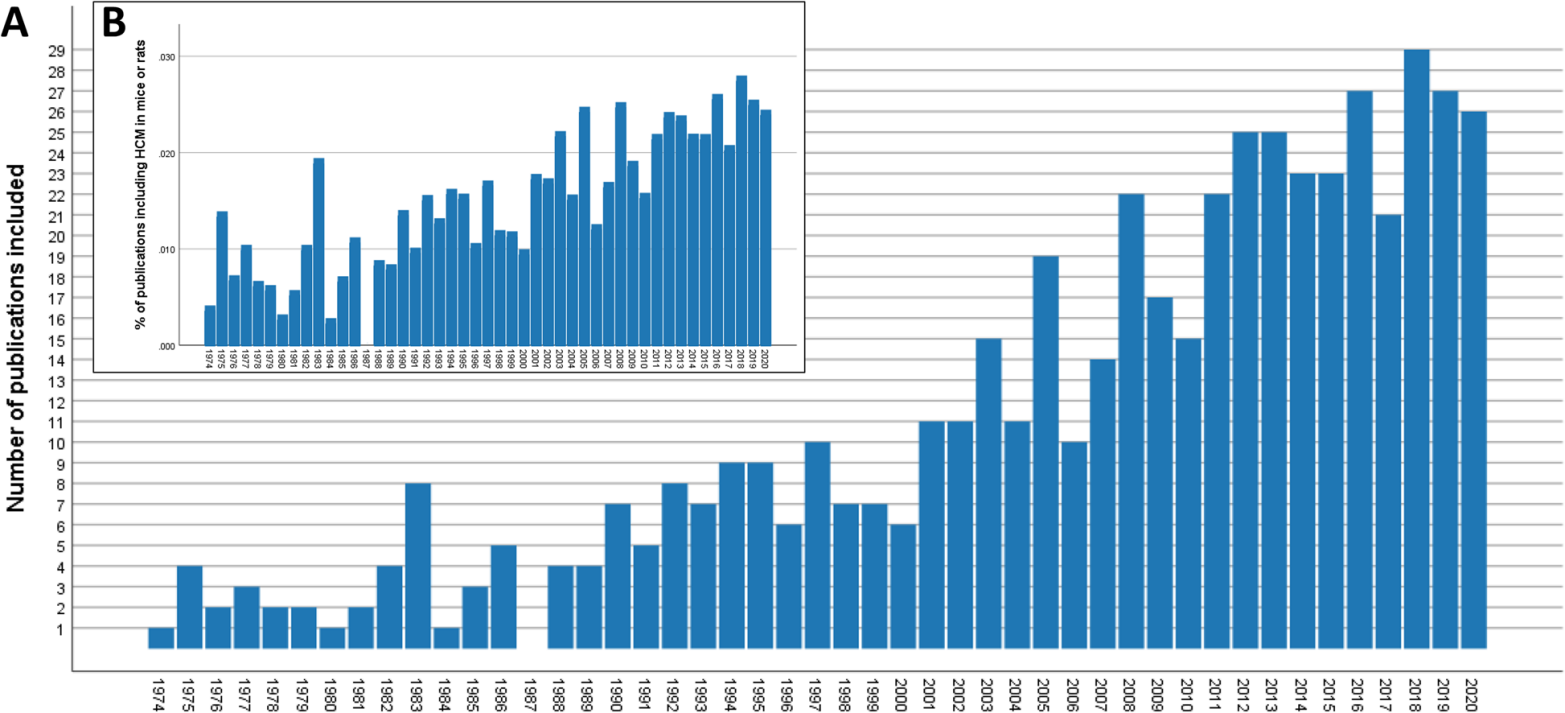
Historical change in the publication rate. **A** Absolute number of included publications on home cage monitoring (HCM) of mice and rats (*n* = 520 publications). **B** The ratio of HCM references (*n* = 520 publications) relative to the overall number of studies in mice or rats (*n* = 3,124,961 publications) published in the years 1974 to 2020. The overall number of studies in mice or rats was retrieved through a search in PubMed using the search string: “mice”[MeSH Terms] OR “mice”[All Fields] OR “rats”[MeSH Terms] OR “rats”[All Fields]; filter: other animals)

The earliest publication fulfilling our criteria for home cage monitoring (Additional file 1) that we could find was published in 1974. In 2018, 2019 and 2020, 29, 27 and 26 publications were identified, respectively. The yearly number of publications in which mice and/or rats were monitored in their home cages according to our eligibility criteria increased (Fig. 2A). In 1987, no publication meeting the inclusion criteria was found. In 2021, only one study fulfilled the eligibility criteria, but since the literature search was carried out in February 2021 the databases did not cover the entire year.

When comparing the number of HCM publications with the overall number of studies in mice or rats listed in PubMed (search string: “mice”[MeSH Terms] OR “mice”[All Fields] OR “rats”[MeSH Terms] OR “rats”[All Fields]; filter: other animals), the percentage of relevant HCM references increased over time (Fig. 2B; linear regression analysis: F(1, 45) = 100.704, *p* < 0.001; R^2^ = 0.691).

### High number of studies using male mice or rats only

**Fig. 3:**
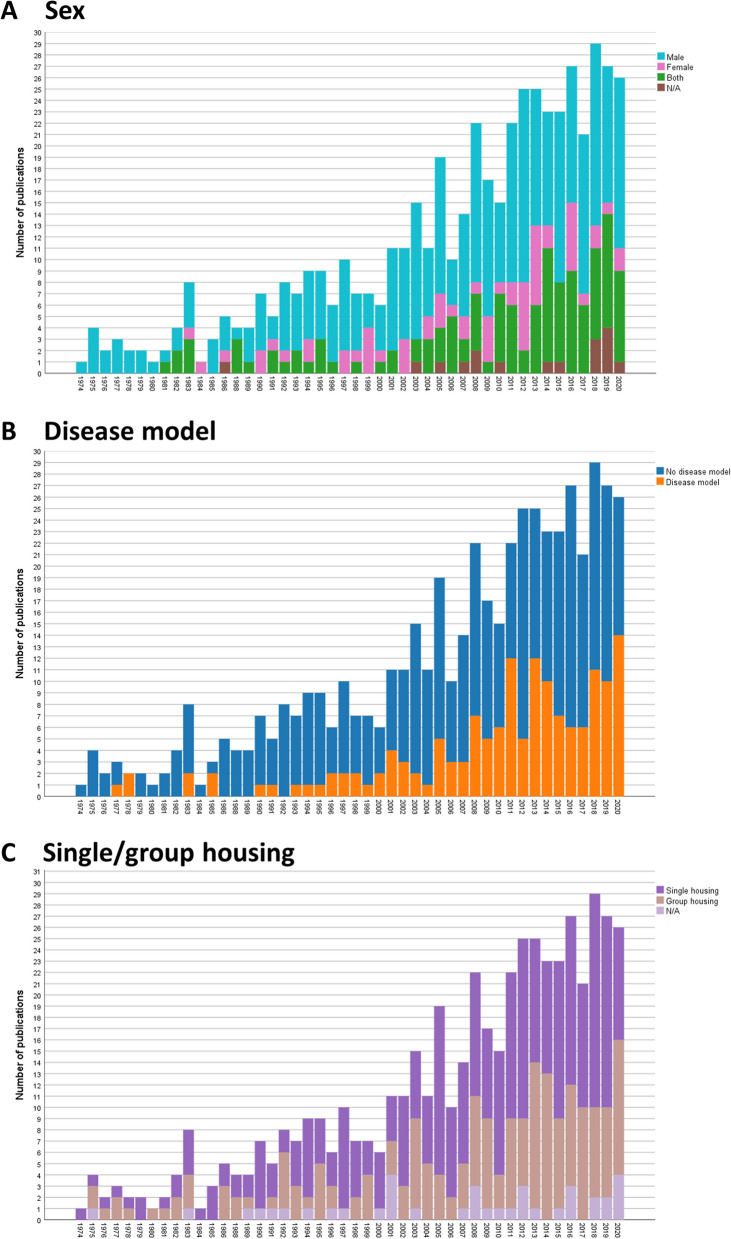
Historical change in the number of publications using male and/or female mice (**A**), publications investigating specific disease models (**B**), and publications employing single or group housing (**C**) (*n* = 520 publications)

Mice were studied in 276 publications, rats in 240, and both species in 5 publications (Table 1). Over all studies, 61% used male animals only. The percentage of studies exclusively using males was 55% in mice (13% females only), 69% in rats (11% females only), and 40% in studies of both species (20% females only). Both sexes were used in 28% of mouse studies, 19% of rat studies, and 20% in studies of both species. For the past 10 years (2011–2020), the percentage of included studies using animals of both sexes increased (Fig. 3A).

**Table 1:** Species and sex of animals used in the included publications

Species and sex	Number of publications				
	**Male**	**Female**	**Both**	**Not indicated**	**All**
Mouse	151	36	77	12	276
Rat	165	26	45	4	240
Both mouse and rat	2	1	1	1	5

### Frequency of strains used

**Fig. 4:**
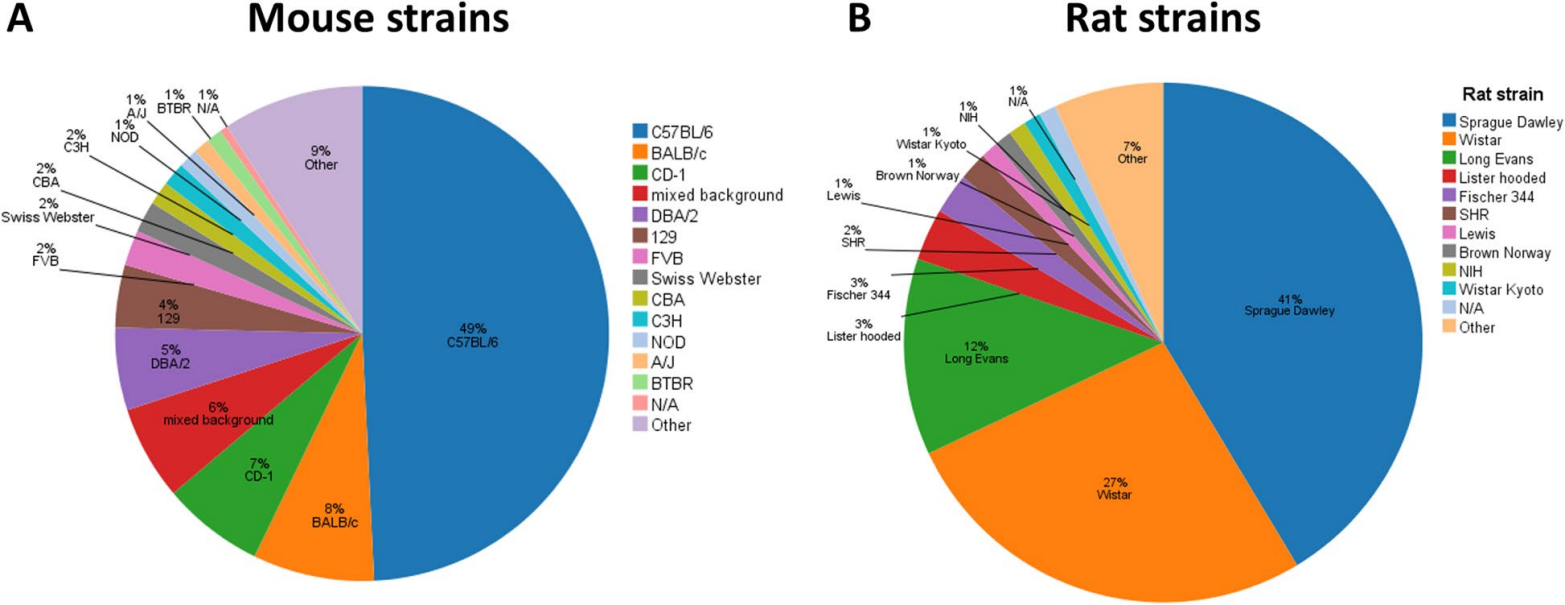
Mouse and rat strains that were used in the included publications in the years 1974 to 2021 (data obtained from *n* = 521 publications). More than one strain could be used in a study. Strains that were indicated twice or once only, were summarized under “other”

The most frequently used mouse strains were C57BL/6 (*n* = 192), BALB/c (*n* = 31), CD-1 (*n* = 26), DBA/2 (*n* = 21), and 129 (*n* = 16), whereas the most frequently used rat stocks were Sprague Dawley (*n* = 115), Wistar (*n* = 74), and Long Evans (*n* = 34) (Fig. 4). In mice inbred strains dominate, whereas in rats outbred strains prevail. In Additional file 2, the numbers of publications including particular strains, even those subsumed under “other” in Fig. 4, are listed.

### HCM studies involving disease models increased within the past two decades

The disease models were categorized according to ICD-11 (https://icd.who.int/browse11/l-m/en). The most frequently studied disease models in the included references were mental, behavioral, or neurodevelopmental disorders (*n* = 74), diseases of the nervous system (*n* = 32), and endocrine, nutritional or metabolic diseases (*n* = 15; Table 2). In a majority of publications—368 references—no disease model was studied. However, the percentage of included studies involving disease models increased in the two past decades (Fig. 3B; 1974–1980: 20%; 1981–1990: 13%; 1991–2000: 18%; 2001–2010: 27%; 2011–2020: 38%).

**Table 2:** Disease models used in the included publications

Disease model	Number of publications
No disease model	368
Mental, behavioral or neurodevelopmental disorders	74
Diseases of the nervous system	32
Endocrine, nutritional or metabolic diseases	15
Diseases of the circulatory system	8
Diseases of the digestive system	5
Diseases of the immune system	4
Infectious or parasitic diseases	4
Developmental anomalies	2
Diseases of the respiratory system	2
Injury, poisoning or certain other consequences of external causes	2
Neoplasms	2
Sleep–wake disorders	2
Symptoms, signs or clinical findings, not elsewhere classified	1
Diseases of the musculoskeletal system or connective tissue	1

In one paper, more than one disease model was studied. The disease models were categorized according to ICD-11 (https://icd.who.int/browse11/l-m/en)

### Growing use of commercially available HCM systems

**Fig. 5:**
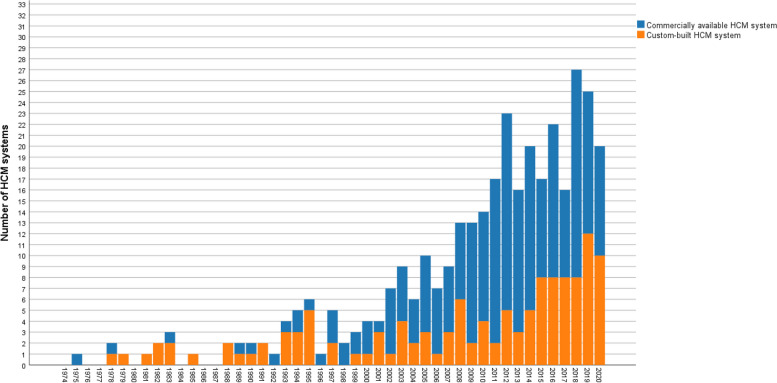
Historical change in the number of custom-built and commercially available home cage monitoring (HCM) systems. More than one system could be applied in a study. In *n* = 126 cases custom-built HCM systems and in *n* = 219 cases commercially available HCM systems were used (multiple HCM systems were applied in *n* = 28 studies). Studies in which no HCM system was used were excluded from this figure

Additional file 3 provides detailed information about the HCM systems used in the included studies. The focus of this table are the techniques and systems specifically developed for the purpose of home cage monitoring. The table contains the number of publications in which the HCM systems were used, and the species, strain/stock, sex, and group size of the animals housed in the systems. Moreover, the duration the animals spent in the particular system is listed. In 126 cases custom-built HCM systems and in 219 cases commercially available HCM systems were used (multiple HCM systems were applied in 28 studies). A “custom-built” system had to fulfill two criteria: (1) it is not commercially available and construction plans and/or software were provided by the authors; (2) the authors gave a name to the system. If these criteria were not met and neither a commercially available nor a “custom-built” system was used, it was considered that no system was applied in the study, e.g., the deployment of a camera only. In 209 publications, no HCM system was used/indicated; in 16 of these 209 publications, a HCM system was used without indicating its name. The use of commercially available HCM systems has increased over time (Fig. 5).

### Single housing remained the most common housing condition

In most studies (57%), animals were individually housed in their home cage system (*n* = 298 publications) (Table 3). In the 1970s, there were as many studies in which animals were kept individually as in groups (Fig. 3C; 1974–1980: 47% group housing, 47% single housing). After 1980, group housing has decreased in comparison to the 1970s, but there was a slight increasing trend between the 1990s and the 2010s (Fig. 3C; 1974–1980: 47%, 1981–1990: 32%, 1991–2000: 31%, 2001–2010: 33%, 2011–2020: 38%). In contrast, the number of studies with single-housed animals has increased after 1980 in comparison to the 1970s and slightly decreased again in the 2000s and 2010s (1974–1980: 47%, 1981–1990: 61%, 1991–2000: 61%, 2001–2010: 59%, 2011–2020: 55%). Table 3 shows that those laboratory rodents that were socially housed were often kept in pairs (*n* = 60 publications) more so than in groups of three (*n* = 27 publications) or four animals (*n* = 30 publications). Other group sizes between five and nine animals were even less frequently found. Groups of ten (*n* = 12 publications) or even more than ten animals (*n* = 16 publications) were rarely reported in the included publications. It must be noted that the indicated numbers were the maximum numbers of animals housed in a cage.

**Table 3:** Maximum number of animals housed together in a cage

Number of animals housed in a home cage system	Number of publications
1	298
2	60
3	27
4	30
5	15
6	9
7	2
8	8
9	6
10	12
More than 10	16
Not indicated	38

Additional file 4 suggests that in single-sex studies individual housing may be more common for male mice than female mice, though this data must be interpreted with caution. For rats, both sexes were predominantly single-housed.

### Animals were mostly kept in the HCM systems for short time periods

**Fig. 6:**
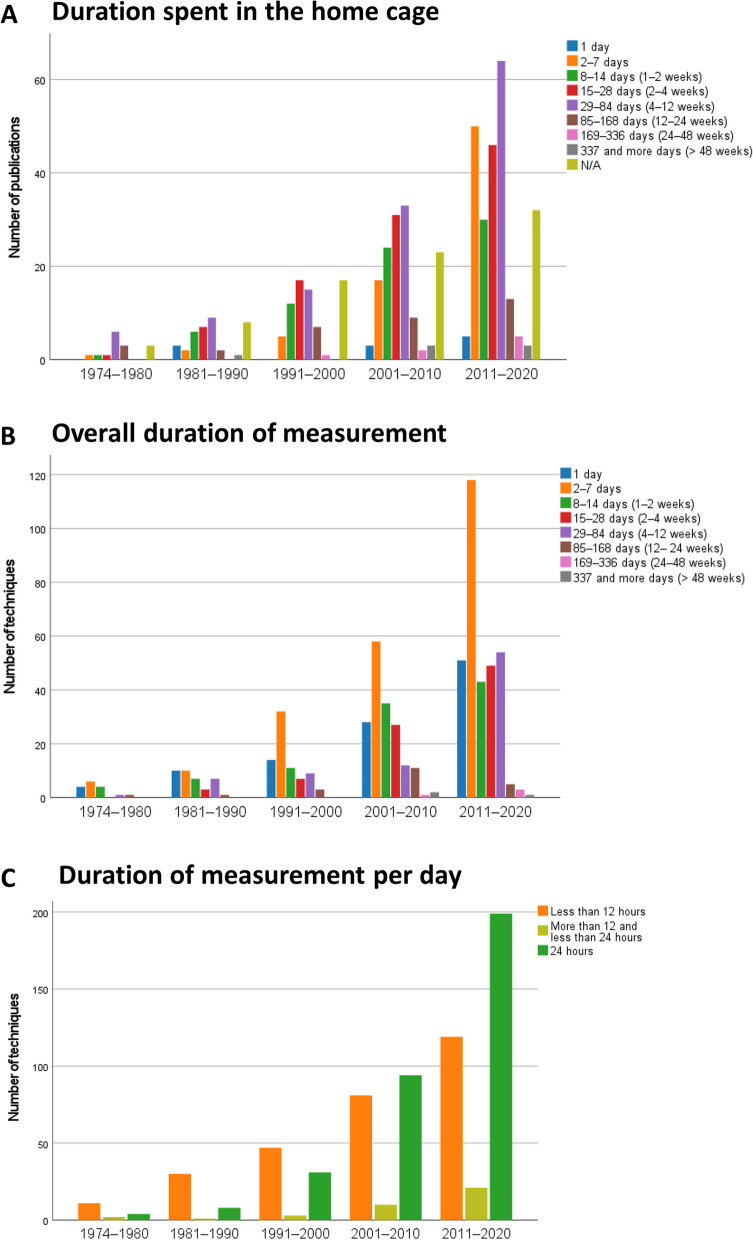
Historical change in the duration of housing and monitoring in the home cage: **A** duration spent in the home cage (*n* = 520 publications), **B** overall duration of measurement (*n* = 667 techniques), **C** duration of measurement per day (*n* = 699 techniques) using a home cage monitoring technique. For **B** and **C**, it must be noted that more than one technique for data measurement could be used in a study. If one of the techniques used to investigate parameters of interests could not be extracted from the publication, this technique was excluded for **B** and **C**. Note the differences in the scaling of the *y*-axis

The duration of housing was classified as short-term (1–28 days), intermediate (1–3 months), or long-term (more than 3 months). Regardless of the publication year, most studies used short-term housing (2–7 days: *n* = 75; 1–2 weeks: *n* = 73; 2–4 weeks: *n* = 102). Intermediate duration housing was not uncommon (4–12 weeks; *n* = 127; Fig. 6A). However, single-day housing for HCM (*n* = 11) and longer periods (12–24 weeks, *n* = 35; 24–48 weeks, *n* = 8; more than 48 weeks, *n* = 7) were rare. In 16% of the studies (*n* = 83), the authors did not indicate the duration of housing.

### Increasing number of studies conducting measurements during the entire time the animals spent in a HCM system

Figure 6B gives information on how long a technique was used for measuring behavioral, physiological, or external appearance–related parameters. In all years, most studies involved measurements of 2–7 days. In 1974–1980, the animals were mostly monitored for 1–14 days. Only in rare cases did the measurements last for 4–24 weeks. In 1981–1990, the proportion of studies in which parameters were analyzed for 2–12 weeks increased. Between 2011 and 2020, the frequencies of measurements over 1 day, 1–2 weeks, 2–4 weeks, and 4–12 weeks were relatively similar, with the latter being clearly increased in comparison to the two past decades. Longer periods of measurement (i.e., > 3 months) were only found in a few cases.

There was a slight change over time in the relation between the housing duration in the HCM systems and the duration for which the parameters of interest were measured. It has become more common to do measurements for the entire period in which the animals are kept in a HCM system over time (1974–1980: 29%; 1981–1990: 44%; 1991–2000: 42%; 2001–2010: 47%; 2011–2020: 54%).

### Duration of monitoring per day in a HCM system increased

As shown in Fig. 6C, in the time period 1974–2000, most measurements lasted for less than 12 h per day. Past the year 2000, 24-h measurements were conducted more frequently.

Overall, automated techniques dominated for measurements of more than 12 h. For monitoring periods of less than 12 h, manual techniques were common (70% of techniques).

### A large range of manual and automatic HCM techniques can be applied for collecting data from group-housed animals

In most studies, one HCM technique was used (*n* = 362). The second most frequently reported option was the combination of two techniques (*n* = 118). A combination of three (*n* = 35) or more (*n* = 6) techniques was less often applied.

Table 4 shows that in a group housing setting, more techniques were applied to measure data from individuals than from groups of animals. Approximately as many manual as automatic HCM techniques were used to generate data from individual subjects. Data from groups of animals were rather collected by manual techniques.

**Table 4:** Percentage of automatic and manual techniques measuring data from individuals and groups of animals in a group-housed setting

	**Automatic **(%, abs. number given in brackets)	**Manual **(%, abs. number given in brackets)
Data from individuals	33 (83)	36 (91)
Data from groups of animals	10 (26)	18 (45)
Data both from individuals and groups of animals	0 (1)	0 (0)
Not indicated	0 (1)	1 (3)

Due to rounding, the sum across the cells does not equal 100%. More than one technique could be applied in a study and the same techniques could be used across the different studies

The techniques listed in Table 5 were used in group-housed animals and almost all of them allowed for generating data for individual animals. However, it must be considered that often two or more techniques were combined, which may allow for collecting data from individuals instead of animal groups only (e.g., by combining a RFID system, which identifies individual animals, with other techniques).

**Table 5:** Percentage of techniques that were applied for measuring data from individuals or groups of animals in a group-housed setting

	**Data from individuals **(%, abs. number given in brackets)	**Data from groups of animals **(%, abs. number given in brackets)	**Not indicated **(%, abs. number given in brackets)
Manual evaluation	64 (87)	34 (46)	1 (2)
Beam-based tracking	72 (13)	28 (5)	–
Visual object tracking (video)^a^	48 (12)	48 (12)	4 (1)
Telemetry	100 (17)	–	–
RFID	92 (23)	8 (2)	–
Electronic sensors and transducers	38 (3)	63 (5)	–
Vibration, force and weight-sensitive tracking	80 (4)	20 (1)	–
Running wheel (counter)	–	100 (2)	–
Drinkometer/Lickometer	100 (4)	–	–
Audio recording	75 (3)	25 (1)	–
Other	100 (6)	–	–

More than one technique could be applied in a study. Due to rounding, the sum across a row does not always equal 100%. ^a^In one study, this technique was applied for measuring both data from individuals and groups of animals

**Fig. 7:**
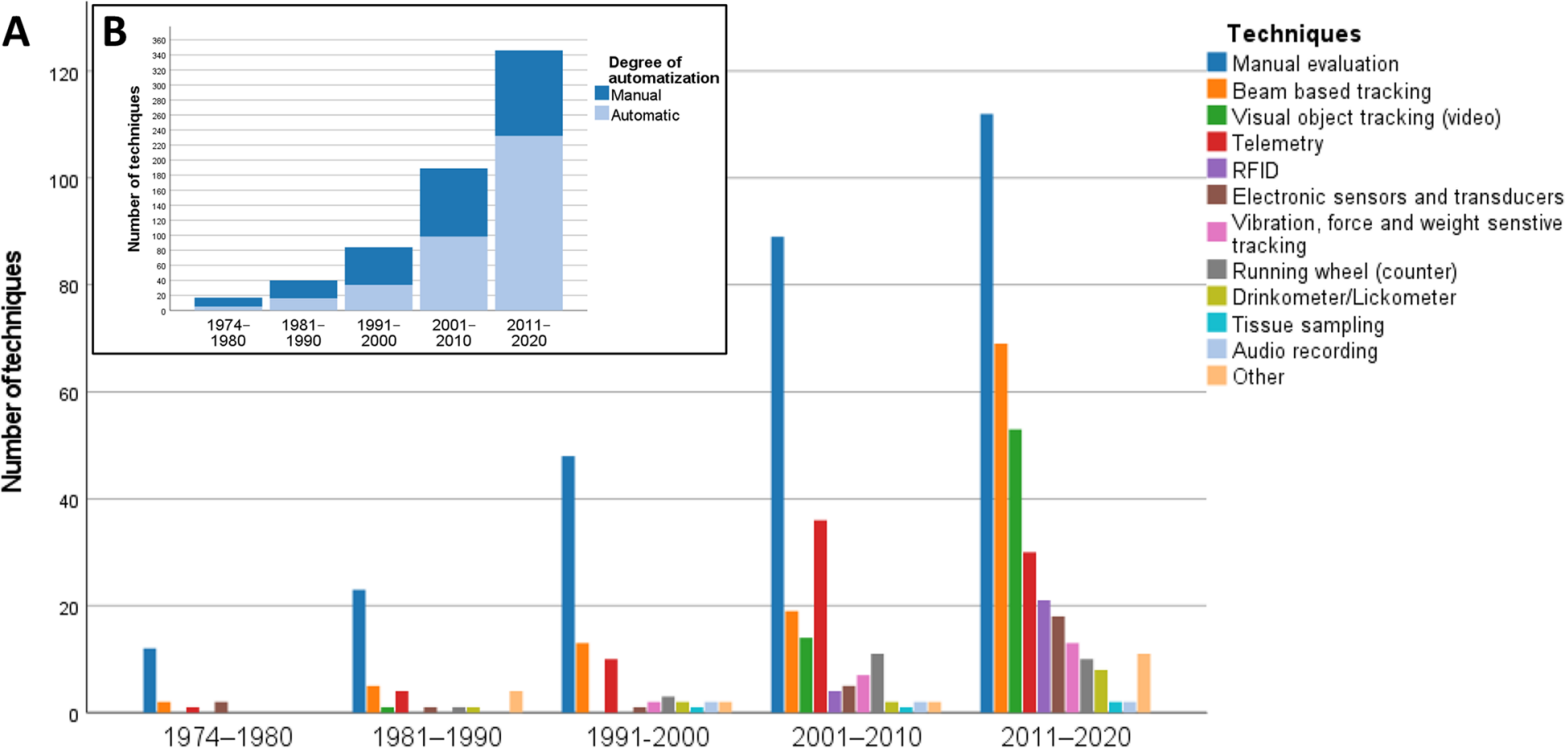
Historical change in home cage monitoring techniques for laboratory mice and rats (data obtained from *n* = 520 publications). More than one technique could be indicated in a publication. If one of the techniques used to investigate parameters of interests could not be extracted from the publication but information on the degree of automatization was given, this technique was excluded for **A** and included for **B**. **A** Number of techniques used in the included publications in the defined time periods. Other: infrared thermometer (*n* = 1), automatic food dispenser (*n* = 1), impedance pneumography (*n* = 1), flowmeter circuit (*n* = 1), lever (*n* = 2), brain imaging cameras (*n* = 2), fiber photometry system (*n* = 1), cardiotachometer (*n* = 1), thermal imaging (*n* = 1). **B** Number of manual and automated techniques that were applied in the publications included

Figure 7A illustrates how the use of techniques for home cage monitoring of laboratory mice and rats has developed over time. Across the whole study periods, manual evaluation (including live monitoring and manual evaluation of videos from RGB or infrared cameras) was the most frequently used technique. In the 2010s, non-invasive methods (i.e., beam-based tracking and visual object tracking) have overtaken the use of telemetry, which is extremely invasive. It should be noted that telemetry and the non-invasive methods mentioned can be automated.

In the periods between 1974–1980, 1981–1990, and 1991–2000, most measurements were performed manually (71%; 57%; 59%) (Fig. 7B). In contrast, between 2001–2010 and 2011–2020 (52% and 68%), most measurements were automated.

In Tables 6, 7, and 8, the techniques are separated into methods for assessing behavior, physiology, and external appearance. Manual evaluation and visual object tracking (video) played a role for almost all behavioral parameters (Table 6). Among other techniques, photo beam (or photo diode)-based tracking and RFID were used to analyze locomotor activity, wheel running, motor and sensory functions, feeding, social behavior, and learning and memory. RFID systems were used for studying spatial preference, and abnormal behavior. Less frequently used techniques can be found in Table 6.

**Table 6:** Techniques used for the analysis of behavioral parameters

Behavioral parameters	Number of indications over all studies
**Locomotor activity** (in 2 studies, 2 techniques were used)	**317**
Manual evaluation	91
Beam-based tracking	81
Visual object tracking (video)	54
Telemetry	48
Electronic sensors and transducers	13
Vibration, force and weight-sensitive tracking	12
RFID (radio-frequency identification)	11
Not indicated	7
**Feeding (drinking, food)** (in 2 studies, 2 techniques were used)	**213**
Manual evaluation	123
Beam-based tracking	21
Visual object tracking (video)	13
Drinkometer/lickometer	11
RFID*	11
Vibration, force and weight-sensitive tracking	9
Electronic sensors and transducers	4
Other (automatic food dispenser)	1
Not indicated	20
**Social behavior**	**117**
Manual evaluation	101
Visual object tracking (video)	11
RFID	2
Audio recording	1
Beam-based tracking	1
Not indicated	1
**Burrowing and nesting**	**56**
Manual evaluation	47
Visual object tracking (video)	3
Beam-based tracking	4
Vibration, force and weight-sensitive tracking	1
Not indicated	1
**Wheel running**	**51**
Running wheel (counter)	25
Electronic sensors and transducers	8
Beam-based tracking	4
Manual evaluation	2
RFID	1
Visual object tracking (video)	1
Not indicated	10
**Abnormal behaviors (e.g., infanticide, barbering, stereotypy)**	**34**
Manual evaluation	31
Electronic sensors and transducers	1
RFID	1
Visual object tracking (video)	1
**Facial expression and body posture**	**29**
Manual evaluation	25
Visual object tracking (video)	4
**Grooming**	**27**
Manual evaluation	20
Visual object tracking (video)	4
Vibration, force and weight-sensitive tracking	3
**Learning and memory**	**25**
Manual evaluation	6
Beam-based tracking	5
RFID	4
Drinkometer/lickometer	2
Electronic sensors and transducers	1
Visual object tracking (video)	2
Other (lever)	2
Not indicated	3
**Anxiety, depression, and schizophrenia**	**15**
Manual evaluation	10
Visual object tracking (video)	4
Not indicated	1
**Sleep behavior**	**12**
Visual object tracking (video)	7
Manual evaluation	3
Telemetry	1
Vibration, force, and weight-sensitive tracking	1
**Vocalization**	**11**
Manual evaluation	6
Audio recording	5
**Motor and sensory functions**	**9**
Visual object tracking (video)	3
Manual evaluation	2
RFID	2
Beam-based tracking	1
Electronic sensors and transducers	1
**Spatial preference**	**6**
Vibration, force, and weight-sensitive tracking	2
Manual evaluation	2
RFID	1
Visual object tracking (video)	1
**Defecation and urination**	**5**
Manual evaluation	4
Not indicated	1
**Sniffing**	**4**
Manual evaluation	3
Visual object tracking (video)	1
**Seizures**	**3**
Manual evaluation	2
Visual object tracking (video)	1
**Other (each *n* = 1)**	

Clinical signs not further specified—manual evaluation; Curiosity/alertness—manual evaluation; Sneezing—manual evaluation; Sign of pain or distress not further specified—manual evaluation; Twitches—visual object tracking (video); Behavior not further specified—visual object tracking (video); Behavior not further specified (“champing behavior”) —manual evaluation

More than one parameter and/or technique could be indicated in a publication and more than one technique could be used to measure a parameter. Note that in some cases, RFID systems are combined with other techniques to identify the animals before a parameter is measured

**Table 7:** Techniques used for the analysis of physiological parameters

Physiological parameter	Number of indications over all studies
**Heart rate & Electrocardiography**	**49**
Telemetry	44
Electronic sensors and transducers	1
Manual evaluation	1
Other (cardiotachometer, non-invasive electrodes)	3
**Body temperature**	**37**
Telemetry	31
RFID	3
Manual evaluation	1
Other (infrared thermometer, thermal imaging)	2
**Body weight**	**2**
Vibration, force, and weight-sensitive tracking	2
**Blood pressure**	**26**
Telemetry	25
Other (flowmeter circuit)	1
**Neuronal activity**	**21**
Telemetry	9
Electronic sensors and transducers	1
Tissue sampling	1
Other (brain imaging cameras, fiber photometry system, implanted electrodes)	9
Not indicated	1
**Respiration**	**8**
Manual evaluation	4
Electronic sensors and transducers	1
Telemetry	1
Visual object tracking (video)	1
Other (impedance pneumography)	1
**Electromyography**	**7**
Telemetry	4
Electronic sensors and transducers	1
Other (implanted electrodes)	1
Not indicated	1
**(Stress) hormones**	**6**
Manual evaluation (from fecal samples)	3
Tissue sampling	3
**Colonic contractility**	
Telemetry	1

More than one parameter and/or technique could be indicated in a publication and more than one technique could be used to measure a parameter. Note that in some cases RFID systems are combined with other techniques to identify the animals before a parameter is measured

**Table 8:** Techniques used for the analysis of external appearance-related parameters

External appearance-related parameters	Number of indications over all studies
**Fur condition**	**6**
Manual evaluation	5
Not indicated	1
**Wounds**	**6**
Manual (live) monitoring	6
**Body Condition (Score)**	**4**
Manual (live) monitoring	4
**Piloerection**	**4**
Manual (live) monitoring	4
**Chromodacryorrhea**	**1**
Manual (live) monitoring	1
**External appearance-related parameters not further specified**	**3**
Manual (live) monitoring	1
Visual object tracking (video)	2

More than one parameter and/or technique could be indicated in a publication and more than one technique could be used to measure a parameter

Telemetry has been used to record most physiological parameters, except for body weight and hormone levels (Table 7). It must be noted that hormone concentrations were measured using microdialysis, automated blood sampling, or in fecal samples. The latter were manually collected from the home cages and analyzed later. In the context of the present systematic review, the collection of fecal samples for subsequent analysis was considered a form of manual evaluation. For the analysis of neuronal activity, telemetry was used in most cases. Respiration was mainly manually assessed. Other techniques such as RFID, electronic sensors and transducers, vibration, force, and weight-sensitive tracking, as well as tissue sampling (e.g., microdialysis) were used less frequently.

External appearance was usually evaluated manually (Table 8).

Detailed information on the methodology of each behavioral, physiological, and external appearance-related parameter (i.e., the degree of automatization, group *versus* individual monitoring, and the duration of measurement) can be found in Additional file 5.

### Behavioral parameters most frequently investigated in the home cage were locomotor activity, feeding, and social behavior

**Fig. 8:**
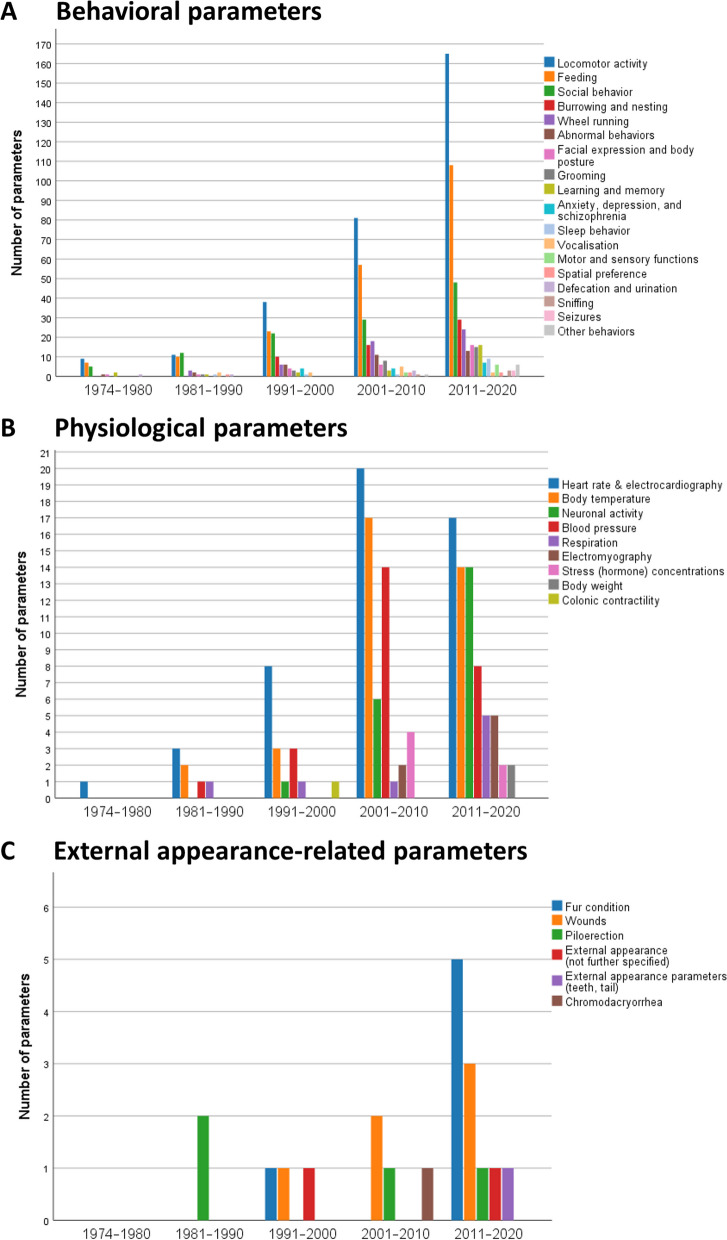
Historical change in monitoring of **A** behavioral parameters, **B** physiological parameters, and **C** external appearance-related parameters in mice and rats in their home cages (data obtained from *n* = 520 publications). More than one application (i.e., parameter) could be indicated in a publication. If the same parameter was investigated using different techniques in a study, the parameter was counted only once for this study. Examples for abnormal behaviors were infanticide, barbering, and stereotypy. Other behaviors (each *n* = 1): sneezing, sign of pain or distress (not further specified), clinical signs (not further specified), curiosity/alertness, champing behavior, twitches, and behavior not further specified. Note the differences in the scaling of the *y*-axis

Figure 8 illustrates the distribution of behavioral, physiological, and external appearance-related parameters between 1974 and 2020. Categories of behavioral parameters (Fig. 8A; a range of behaviors were subsumed under a category) that have been investigated throughout were locomotor activity, feeding, social behavior, abnormal behavior, facial expressions and body posture, as well as learning and memory—with locomotor activity, feeding, and social behavior being the most frequently examined parameters. Locomotor activity stands out in particular. While defecation and urination were already examined in the 1970s, wheel running, grooming, vocalization, sleep behavior, and spatial preference were studied in the home cage for the first time in the 1980s. Observations of burrowing and nesting, or other home cage behaviors used in the modelling of human disorders such as anxiety, depression, and schizophrenia were first reported in the 1990s. Sniffing as well as motor and sensory functions became of interest in the 2000s. Seizures were monitored in the home cage for the first time in the 2010s.

### Physiological parameters most frequently investigated in the home cage were heart rate and/or electrocardiography

Among the physiological parameters (Fig. 8B), heart rate and/or electrocardiography were the most popular and were reported in all periods. The analysis of body temperature in the home cage became relevant in the 1980s. Its use has increased over time. It was the second most frequently examined physiological parameter in the 2000s and 2010s. Blood pressure and respiration have been measured in the home cage since 1983, neuronal activity (including electroencephalograms from implanted electrodes) since 1998, and body weight since 2017. The analysis of electromyography and hormone concentrations in the home cage was first reported between 2001 and 2010; but even later, they were investigated in a few studies only. In 2001–2010, blood pressure was the third most popular physiological parameter; in 2011–2020, neuronal activity was examined more often than blood pressure.

### External appearance-related parameters were rarely investigated in the home cage

External appearance-related parameters have been investigated since 1988 but very few studies focused on these aspects as readouts (Fig. 8C).

## Discussion

In the present systematic review, based on 521 references retrieved through PubMed and Web of Science (until Feb 2021), we studied yearly changes in HCM of laboratory mice and rats between 1974 and 2020. We found an increase in the use of HCM over time. The number of studies using animals of both sexes has grown, as has the study of specific disease models since the last decade(s). Over time, novel HCM techniques have been introduced and the degree of automatization has increased, allowing monitoring of more challenging animal-based parameters in the home cage. Moreover, longer housing in HCM systems with continuous monitoring, also under social housing conditions, are some of the key developments we were able to note with respect to the monitoring of mice and rats in their home cages.

### From heterogeneity to harmonization of the home cage definition

The home cage definition played a central role for the present systematic review since references were only included if the following criteria were met: a home cage must allow animals to be housed permanently in their familiar social structure. We considered it important that the structure should not be changed for monitoring the parameters of interest, since this could influence the data and compromise animal welfare [16, 19]. However, animals that were kept individually throughout the process of monitoring also met our home cage definition. This definition excluded all studies in which group-housed animals were separated for the purpose of testing.

The definition of a home cage varies in the literature and may change over time with new scientific findings and ethical norms. For instance, the definition by Baran et al. focused on the time the animals spent in the “cages […] where animals are housed for the majority of their lifetime in the vivarium”, i.e., their home cage [27]. In contrast, the definition of the present systematic review concentrated on the social structure.

To harmonize the definition of a home cage, experts in the field of HCM recently made efforts to develop a more detailed definition: www.cost-teatime.org/about/hcm-definition. This definition is structured as an olog (i.e., a categorical framework for knowledge representation) [59]. It can be used for checking whether a system fulfills the criteria of HCM and it may serve as a future reporting guideline for HCM systems. However, it must be noted that the HCM definition for this systematic review was developed before the ongoing discussion on terminology and could not be updated after registration of the systematic review protocol.

### Limitations due to search term, exclusion criteria, and date of search

Since the search term used in the present systematic review contained the term “home cage” but no alternative spellings or descriptions except from “housing cage”, some HCM articles may have been missed. For example, publications that did not explicitly focus on HCM and therefore did not use this term, may have not been identified by our search strategy. Moreover, HCM may no longer be mentioned in articles when it has already become a routine procedure in a laboratory and considered as standard. Additionally, the terminology of HCM can depend on the research field. For example, researchers studying natural behavior may use the term “home cage monitoring,” while other researchers assessing learning in the home cage may call it “automated group-housed learning”.

However, in the absence of a harmonized definition of “home cage,” the lack of consensus on the use of descriptors and key words, and the widespread use of HCM in a range of research fields, a comprehensive search of HCM publications would have been nearly impossible.

Besides the home cage definition and terminology, the inclusion and exclusion criteria limited the number of studies that were considered. Studies involving only calorimetric measurements in the home cage were excluded since the animals usually must be transferred to a metabolic cage where they are individually housed for a short term (i.e., they are separated from their social group). Due to this exclusion criteria, only those metabolic studies were included in which also other parameters (e.g., locomotor activity) than metabolic outcomes were investigated.

### Publication rate

The absolute number of publications in which mice and/or rats were monitored in their home cages appeared to increase from 1974 to 2020, especially around 2005 and in the following years. Around 2005, instrumental “home cage-like” test cages were brought to the market [6, 27, 60]. A few years later, home cages with capacitance sensors, RFID, and/or video tracking were developed [27]. The results of our systematic review demonstrated that the use of commercial systems has increased considerably. Custom-built systems were frequently used until the 1990s. Since the 2000s, it appeared that more commercial than custom-built systems were used. According to our definition of custom-built systems, in 209 studies no system was used or indicated. In 16 of these 209 publications, the name of a HCM system that seemed to have been used was not indicated which does not allow to conclude whether a commercial or custom-built system was applied.

The high proportion of commercially available HCM systems used in recent years suggested that researchers increasingly monitored animals in their home cages since HCM systems were available on the market and more easily accessible. However, custom-built set-ups remained important tools, indicating that the HCM field is still in its early development and commercial systems currently cannot fully satisfy the demand of the user community. Another reason for the popularity of custom-built systems may be that open-source custom-built systems are more transparent and allow for faster development and adoption of the techniques. This is in line with the move towards open science and improving reproducibility. In future work, it may be of interest to determine whether commercial and custom-built HCM systems are used to investigate parameters of different complexity. It may be hypothesized that “simple” parameters are investigated using commercial HCM systems, but custom-built systems are required for more complex parameters. However, the HCM systems were not assigned to the parameters investigated in the present systematic review, and therefore the results do not provide this information.

In relation to the total number of PubMed-listed studies including mice or rats, the percentage of HCM publications increased over time. The overall annual publication growth rate in Life Science is 5% according to Bornemann et al. [61].

### Sex bias

We found a strong male-bias for both mice and rats, as reported previously for non-human mammals in other fields of biomedical research [62]. Beery and Zucker evaluated journal articles from 2009 and found a male skew in “neuroscience, physiology, pharmacology, endocrinology, and zoology” [62]. Flórez-Vargas et al. revealed a strong male skew in some mouse models, such as cardiovascular disease models, between 2001 and 2014 [63]. Male animals were often preferred by researchers intending to reduce potential higher data variability related the different phases of the estrous cycle in females [62]. However, the evidence for this is contentious [64–68]. A female bias was found in reproduction and immunology [62], and infectious disease mouse models [63]. Approximately 15% of studies published in the Journal of Physiology (London) and the Journal of Pharmacology and Experimental Therapeutics between 1909 and 2009 used non-human mammals of both sexes [62]. This percentage was a little higher (24% over all years) in the studies included in our systematic review and, interestingly, increased since the 2010s (29%)—probably due to a growing awareness of the consequences of a sex bias in animal-based research [69]. Finally, we would like to note the considerable advantages of testing both male and female rodents in basic research studies to better explore diseases which display variable prevalence in males and females.

### HCM allows social housing

Under natural conditions, the house mouse (*Mus musculus*) lives in relative stable social groups of up to 10 mice/m2. The social structure is a despotic dominance hierarchy with a dominant male, several subordinates, and several females and their offspring. Rank fights may occur between males and may result in the death of a male. A dominance hierarchy is also established among females, in which only the dominant females reproduce. Rats, on the other hand, live in larger colonies with several hundred individuals of both sexes. They live in polygenic societies with promiscuous mating and low agonistic behavior (reviewed in [70]). The conditions under which laboratory animals are kept vary widely.

Our systematic review revealed that male mice and rats of both sexes were individually housed in the majority of studies, although individual housing can impair the emotional state of the animals. In mice, social isolation is known to increase anxiety-related behavior and depressive states [71, 72], to elevate corticosterone concentrations and reduce levels of brain-derived neurotrophic factor (BDNF) [73]. Moreover, social deprivation was shown to affect how mice react to a stressor [74]. Social deprivation is associated with welfare concerns and can result in changes in behavioral, physiological, and neurochemical parameters, metabolism, brain structures, and processes [75–77]. In neurodegenerative mouse models, single housing can affect the disease progression [78]. According to our systematic review, the number of studies in which laboratory rodents were individually housed has slightly decreased in the 2000s and 2010s when compared to the previous two decades. This may also be due to the technological progress in the last decades and availability of HCM systems allowing group housing. However, because of inter-individual aggression with the onset of sexual maturity, male mice are often separated from their same-sex cage mates to avoid stress, and injuries [79–81]. It is worth noting that aggressive behavior in group housed male mice seems also to be affected by the group and cage size [82, 83]. In the studies included in our systematic review, female mice, in contrast to male mice, were more likely to be kept in groups. This may be explained by the feasibility of group housing for females.

The ability to obtain individual data from group-housed animals allows the housing conditions to be adapted to the needs of the animals. Furthermore, depending on the system and group size, several animals could be tested at the same time, which could counteract a possible batch effect. However, the development of the social structure under laboratory conditions, including available space and environmental enrichment in the HCM system, must be taken into account when interpreting the data.

### Duration of housing and monitoring in the home cage

In most studies included in our review, the laboratory rodents were kept for periods of 2 days to 3 months in the HCM systems. It should be noted that data generated from animals that were only kept in the system for a few days before measurement may be biased by the novel environment and/or handling. If animals are transferred from another cage to a HCM system, it can take them a while to adapt to this novel environment. The transfer to a HCM system is comparable to a cage change. The olfactory cues as well as the familiar visual and tactile environments are removed and replaced by a clean cage, bedding, nest material, and enrichment items, which is potentially stressful for animals that strongly rely on scent for communication [84]. This can influence behavioral and physiological parameters [27, 46, 85], such as sleep [86], activity patterns [46], breathing rate [85, 87], heart rate and mean arterial blood pressure [88, 89], and corticosterone concentrations [90]. Since the animals usually are handled when cages are changed, the effects listed above may not only be due to a novel environment but can also be associated with the handling techniques used [18, 91].

Since habituation times are rarely reported, we refrained from extracting the time the animals were allowed to acclimatize to the respective HCM. Instead, data on the duration of measurement using the HCM techniques were collected. HCM techniques were mainly applied for short-term periods (i.e., 1–28 days). The number of techniques being applied for intermediate time periods (i.e., 4–12 weeks) slightly increased over time in the years between 2011 and 2020; however, 2–7 days remained the most frequent duration of measurement. Only in rare cases, HCM techniques were applied for long-term periods (i.e., more than 3 months), meaning only few longitudinal studies were carried out. However, the number of studies involving measurements during the entire time in which the animals were kept in the HCM systems slightly increased over the years, which may relate back to the development of data capture, storage, and analysis techniques.

### Towards 24/7 automated home cage monitoring

HCM techniques were increasingly used for continuous data recording. Our systematic review provides evidence that the development towards 24/7 surveillance has gone hand in hand with automatization. Since the 2000s, a majority of applied HCM techniques have collected data automatically, and measurements have been recorded for 24 h a day. Long-term automated 24/7 surveillance of the animals in their home cage will allow for unbiased measurements.

An important observation is the inter-individual variation in spite of genetic homogeneity and strict standardization of husbandry and experimental conditions. This inter-individual variation can also be observed in HCM studies. It has been shown that differences in activity are more stable over time, which allows more predictability [49, 92–94]. In HCM studies, individuals can be observed over a longer period of time, which can lead to a better understanding of the increased susceptibility or decreased resilience of subpopulations. This is a major advance for phenotyping, monitoring of effect size of interventions, and animal welfare.

Twenty-four hours HCM takes into account the normal circadian rhythm of behavioral and physiological parameters and will reveal any phase shift relative to the light–dark-cycle (for review, see [95]). When nocturnal animals such as mice or rats are monitored for a few hours only, and then often during the light phase, valuable information may be missed, as Eikelboom and Lattanzio showed for running wheel activity [15, 96].

Automatization allows for monitoring of animals without the presence of an experimenter. Prey animals such as mice and rats may show altered behavior and hide for instance pain, suffering, or distress [97]. Therefore, live cage-side health checks may not detect impaired well-being. Automated systems can thus be an important supplement to professional visual inspection of animal health and well-being. However, in our view, the cage-side checks performed by the animal care staff should never fully be replaced since there is always the risk of technical failures. It is widely discussed that an experimenter can influence animal-based parameters [98]. For example, the odor of male experimenters was shown to cause stress and stress-induced analgesia in mice and rats [99]. However, a recent multi-laboratory study demonstrated that experimenters caused less data variation than the different laboratories [100].

In contrast to manual evaluation, automatization allows for more unbiased and data-driven assessment of parameters, which can save labor time once an automated method is established.

### Use of novel techniques allows for monitoring sophisticated parameters

Between 1974 and 1980, besides the manual evaluation of videos or live observations, beam-based tracking [101, 102], telemetry [40], and electronic sensors and transducers [103, 104] were the first techniques applied for HCM. Interestingly, manual evaluation is still relevant in recent times and was the most frequently used technique in all time periods. The role of beam-based tracking, telemetry, and RFID systems became more and more important over time. Moreover, visual object tracking (video) has experienced a major boost (i.e., an increase in use) since the 2010s. As data processing technology has advanced, an increased number of data parameters can be collected in the home cage.

In the 2010s, there was a shift in technology with non-invasive methods (i.e., beam-based tracking and visual object tracking) overtaking invasive methods (i.e., telemetry), which may indicate a shift towards improved animal welfare. The use of implanted telemetry devices compromises animal welfare. Thus, in the interest of animal welfare, non-invasive techniques should be preferred over those that are associated with a burden on the animals [105]. Although not all parameters measured by telemetry can be analyzed using beam-based tracking and visual object tracking, activity can be monitored using non-invasive methods. Moreover, non-invasive alternatives such as jacketed telemetry could be applied for assessing respiration, collecting an electrocardiogram, or measuring activity [106].

Most studies conducted so far with HCM (69%) used only one technique. However, studies collecting a larger set of parameters or extracting data from individuals among group-housed animals usually deployed multiple techniques to increase the versatility of the HCM system. The present systematic review revealed an increase in the number of HCM studies involving disease models, which may be attributed to the technical progress to examine more and more parameters in the home cage and the demand to characterize a rapidly growing number of genetic mutants since the 2000s.

External appearance-related parameters (e.g., body condition score, wounds, fur condition, piloerection, chromodacryorrhea) have only rarely been quantified in the home cage yet although they are crucial for tracking the animals’ welfare status. We envision that machine learning approaches will play a central role in HCM data processing and in the future may also enable automatic monitoring of external appearance-related parameters. Unsupervised and supervised machine learning can be used to automatically analyze video frames recorded in the home cage [107–109].

## Conclusions

All in all, our systematic review revealed that HCM in mice and rats has gone through a considerable development and, as these instruments have become more comprehensive, easy to apply, and scalable, the use of HCM has increased. There is a slight trend towards recordings covering 24 h over intermediate time periods as the HCM techniques are refined and become more and more automated, and application of HCM is spreading to a wider range of study types. A considerable fraction of the HCM systems is still custom-built, but commercial “key ready” solutions are increasingly available and since the 2000s more than 50% of the HCM studies used commercial products.

Although manual observations made live or from videos remain key technical solutions for HCM, this review indicates that a number of alternative, high-throughput, and man-power saving techniques have been introduced.

Future inventions will pave the way for continuous non-invasive rodent monitoring in longitudinal studies. Storage and analysis of the large amounts of data generated by automated HCM are a bottleneck that needs to be addressed [27]. Moreover, the financial aspects may be a hurdle in implementing commercial systems. Nevertheless, the development of new recording and analysis methods is rapid and, moreover, many of these are freely available. We, therefore, see a paradigm shift towards more and more home-cage based methods for the coming research.

## Methods

### Review protocol

The protocol of the systematic review was uploaded to the Open Science Framework on May 3, 2021, after the search for literature was conducted: https://osf.io/4gzcx. It was registered on March 14, 2022 when the full text review and extraction phase were in progress: https://osf.io/un5e6.

### Home cage definition

A home cage was defined as any cage in which the animals could potentially be housed permanently in their familiar social structure (i.e., group or single housing). A testing apparatus could be connected to the home cage allowing the animals to voluntarily enter it. In this scenario, the animals may also separate themselves from the group when entering the testing apparatus. The home cage could be permanently or temporarily divided so that the animals are separated from each other by a cage divider (e.g., a grid). Our home cage definition excluded any cage system that required the separation of group-housed mice or rats for the duration of monitoring. In contrast, if an animal was permanently kept socially isolated in a cage system, the home cage definition was met.

### Search strategy and screening

Primary databases were searched through PubMed and Web of Science on February 24, 2021. PubMed search terms were refined using the search refiner tool QueryVis [110]. The following search strings were used for obtaining relevant studies.

PubMed: ((“mice”[MeSH Terms] OR mice OR mouse) OR (“rats”[MeSH Terms] OR rat OR rats)) AND ((home cage) OR (housing cage)) AND (monitoring OR (“observation”[MeSH Terms] OR observation)). The filter “other animals” was applied.

Web of Science: ALL FIELDS: (((mice OR mouse) OR (rats OR rat)) AND ((“home cage”) OR (“housing cage”)) AND (monitoring OR observation)).

The data extraction template can be found in Additional file 6. Further detailed information on the objective and materials and methods is provided in Additional file 1. In brief, only primary studies (published in English language) in which behavioral, physiological, and/or external appearance-related parameters were monitored in mice and/or rats in their familiar social structure in their home cage or a testing apparatus connected to the home cage were included. The definition of a home cage can be found in Additional file 1. Studies in which no other parameters than calorimetric measurements were examined in a home cage were excluded. In phase 1, titles and abstracts were screened. Thereafter, full texts were screened (phase 2) and data were extracted (phase 3) simultaneously. In all phases, all papers were screened by two independent reviewers and discrepancies were resolved by a third reviewer.

### Analysis

The responses for the mouse/rat strain were reviewed by two persons: In mice, the 129 substrains were subsumed under “129,” C57BL/6 J and C57BL/6N under “C57BL/6,” and ICR and CD-1 under “CD-1.” In rats, Holtzman and Sprague Dawley were subsumed under “Sprague Dawley.” For genetically modified animals, the background strain was extracted. If strains were of a mixed genetic background, “mixed background” was indicated.

If responses appeared not to be plausible or free text-responses had to be clustered, the answers were corrected and the corrected responses were used for further analysis. All original and corrected responses can be found in Additional file 7.

SPSS (IBM Corp. Released 2020. IBM SPSS Statistics for Windows, Version 27.0. Armonk, NY: IBM Corp) was used for the linear regression analysis and for creating the figures. The year 2021 was excluded in the figures visualizing the historical change since the literature search was carried out in February 2021 and did not cover the entire year. The ratio of relevant HCM references per year was analyzed using a linear regression model. The R^2^ (fraction of explained variance) indicates how well the model fits the data (from 1, best prediction to 0, no prediction). The remaining data were descriptively analyzed.

### Supplementary Information

**Additional file 1.** Objective, Materials & Methods.

**Additional file 2.** Mouse and rat strains (more than one strain could be used in a study; data obtained from *n* = 521 publications.

**Additional file 3.** Home cage monitoring system (more than one system could be used in a study; data obtained from *n* = 521 publications).

**Additional file 4.** Social housing structure in single-sex studies (data obtained from *n* = 187 single-sex studies including mice and *n* = 191 single-sex studies including rats).

**Additional file 5.** Behavioral, physiological, and external appearance-related parameters (data obtained from *n* = 521 publications).

**Additional file 6.**  Data extraction template.

**Additional file 7.** Data (data obtained from *n* = 521 publications).

## Data Availability

All data generated or analyzed during this study are included in this published article and its supplementary information files. The dataset can be found in Additional file 7.

## Abbreviations

TEATIME COST Action: “Improving biomedical research by automated behaviour monitoring in the animal home-cage”
HCM: Home cage monitoring
LED: Light-emitting diodes
RFID: Radio-frequency identification
