# Supplementary material for: A systematic review of the development and application of home cage monitoring in laboratory mice and rats
Source: BMC Biol. 2023 Nov 13;21:256. doi: 10.1186/s12915-023-01751-7 (PMC10642068; doi:10.1186/s12915-023-01751-7)
Supplement: Supplementary file 3 — Additional file 3. Home cage monitoring system (more than one system could be used in a study; data obtained from n = 521 publications). [file 12915_2023_1751_MOESM3_ESM.docx]

The most frequently used commercially available HCM systems were the Data Sciences International telemetry devices (DSI, USA; n = 48). Other telemetry devices were produced by Starr Life Sciences (USA, E-Mitter Telemetry Implants, n = 7), Mini-Mitter (USA, n = 2), and Transoma Medical (USA, telemetry probe, n = 1).

A range of running/activity wheels from different companies have been used: Lafayette Instruments (USA, n = 4), Mini-Mitter (USA, n = 2), MED Associates (USA, n = 2), ClockLab, Actimetrics (USA, n = 2), TSE (USA/Germany; n = 2), Wahmann Mfg. Co., Model LC-34, n = 1), Tecniplast (Italy, n = 1), Shinano Ltd. (Japan, n = 1), Respironics (USA, n = 1). In one study, the manufacturer was not indicated.

Other HCM systems found in publications were the Phenotyper (Noldus, the Netherlands; n = 26), HomeCageScan (Cleversys, USA; n = 10), IntelliCage (TSE, USA/Germany; n = 9), PhenoMaster (TSE, USA/Germany; n = 7), InfraMot (TSE, USA/Germany; n = 6), DVC (Tecniplast, Italy; n = 5), HCA (Actual analytics, United Kingdom; n = 5), Laboras (Metris, the Netherlands; n = 4), EthoVision (Noldus, the Netherlands; n = 4), Activity monitor (AccuScan Instruments, USA; n = 4), Opto-M3 Dual Axis System (Columbus Instruments, USA; n = 4), Operant chambers (MED Associates, USA; n = 3), ActiviScope (NewBehavior, Switzerland; n = 3), 24 channel activity monitoring system (O'Hara & Co., Tokyo, Japan; n = 3), Home Cage Activity System (Coulbourn Instruments, Allentown, PA, USA; n = 3), operant chambers (Coulbourn Instruments, USA; n = 2), MicroMax Activity monitor (AccuScan Instruments, USA; n = 2), Animex activity meter (LKB Instruments, Sweden; n = 2), and Infrared Motion Detector (Starr Life Sciences, USA; n = 2).

| **Home cage monitoring systems** |  | **Number of publications** | **Species (multiple answers possible)** | **Strain (multiple answers possible)** | **Sex** | **Number of animals housed in the home cage system** | **Duration spent in the home cage system** |
| --- | --- | --- | --- | --- | --- | --- | --- |
| N/A (no system used/indicated) |  | 209 |  |  |  |  |  |
| More than one system |  | 28 |  |  |  |  |  |
| Custom-built |  | 126 |  |  |  |  |  |
| **Telemetry** |  |  |  |  |  |  |  |
| Telemetry Devices (DSI, USA) |  | 48 | Mouse (n=28)  Rat (n=20) | C57BL/6 (n=17)  CD-1 (n=4)  BALB/c (n=3)  FVB (n=2)  DBA/2 (n=1)  Swiss SE (n=1)  Mixed background (n=2)  Sprague Dawley (n=10)  Wistar (n=9)  SHR (n=3)  BDIX (n=1)  Brown Norway (n=1)  Fischer 344 (n=1)  Wistar Kyoto (n=1) | Male (n=36)  Female (n=7)  Both (n=4)  N/A (n=1) | 1 (n=34)  2 (n=5)  3 (n=2)  4 (n=1)  N/A (n=6) | 1 day (n=1)  2–7 days (n=5)  8-14 days (1–2 weeks) (n=8)  15-28 days (2–4 weeks) (n=12)  29-84 days (1–3 months) (n=7)  85-168 days (3–6 months) (n=4)  N/A (n=11) |
| E-Mitter Telemetry Implants (Starr Life Sciences, USA) |  | 7 | Mouse (n=5)  Rat (n=2) | C57BL/6 (n=3)  DBA/2 (n=1)  NIH Swiss (n=1)  SHR (n=1)  Sprague Dawley (n=1)  Wistar Kyoto (n=1) | Male (n=5)  Female (n=2) | 1 (n=4)  2 (n=1)  N/A (n =2) | 2–7 days (n=1)  8-14 days (1–2 weeks) (n=3)  15-28 days (2–4 weeks) (n=2)  29-84 days (1–3 months) (n=1) |
| Telemetry probe (Transoma Medical, USA) |  | 1 | Rat (n=1) | Sprague Dawley (n=1) | Male (n=1) | 1 (n=1) | 2–7 days (n=1) |

| Telemetry (Mini-Mitter, USA) | |  | 2 | | Mouse (n=2) | | C57BL/6 (n=1)  AKR (n=1)  SWR (n=1)  db/db (n=1) | | Male (n=2) | | 1 (n=2) | | N/A (n=2) | |
| --- | --- | --- | --- | --- | --- | --- | --- | --- | --- | --- | --- | --- | --- | --- |
| **Wheel running** | |  |  | |  | |  | |  | |  | |  | |
| Running wheels (Mini-Mitter, USA) | |  | 2 | | Mouse (n=2) | | C57BL/6 (n=1)  Mixed background (n=1) | | Male (n=1)  Both (n=1) | | 1 (n=1)  4 (n=1) | | 8–14 days (1–2 weeks) (n=1)  N/A (n=1) | |
| Running wheels (MED Associates, USA) | |  | 2 | | Mouse (n=1)  Rat (n=1) | | C57BL/6 (n=1)  Sprague Dawley (n=1) | | Male (n=2) | | 1 (n=2) | | 8–14 days (1–2 weeks) (n=1)  29–84 days (1–3 months) (n=1) | |
| Activity wheels (Wahmann Mfg. Co., Model LC-34) | |  | 1 | | Rat (n=1) | | Wistar (n=1) | | Male (n=1) | | 1 (n=1) | | 15–28 days (2–4 weeks) (n=1) | |
| Activity wheels (Lafayette Instruments, USA) | |  | 4 | | Mouse (n=3)  Rat (n=1) | | C57BL/6 (n=3)  Long Evans (n=1) | | Male (n=2) Both (n=2) | | 1 (n=2)  N/A (n=2) | | 15–28 days (2–4 weeks) (n=1)  29–84 days (1–3 months) (n=1)  85–168 days (3–6 months) (n=1)  169–336 days (6–12 months) (n=1) | |
| Running wheels (manufacturer not indicated) | |  | 1 | | Rat (n=1) | | Sprague Dawley (n=1) | | Female (n=1) | | 1 (n=1) | | 85–168 days (3–6 months) (n=1) | |
| Running wheels (ClockLab, Actimetrics, USA) | |  | 2 | | Mouse (n=1)  Rat (n=1) | | Mixed background (n=1)  Wistar (n=1) | | Male (n=1)  Both (n=1) | | 1 (n=2) | | 2–7 days (n=1)  29–84 days (1–3 months) (n=1) | |
| Running wheels (Tecniplast, Italy) | |  | 1 | | Rat (n=1) | | Wistar (n=1)  Sprague Dawley (n=1) | | Male (n=1) | | 2 (n=1) | | 29–84 days (1–3 months) (n=1) | |
| Running wheels (TSE, USA/Germany) | |  | 2 | | Mouse (n=2) | | C57BL/6 (n=2)  NZB (n=1)  NZO (n=1) | | Male (n=1)  N/A (n=1) | | 1 (n=2) | | 2–7 days (n=1)  N/A (n=1) | |
| Running wheels (Shinano Ltd, Japan) | |  | 1 | | Rat (n=1) | | Wistar (n=1) | | Male (n=1) | | 1 (n=1) | | 8–14 days (1–2 weeks) (n=1) | |
| Running Wheels (Respironics, USA) | |  | 1 | | Mouse (n=1) | | CD-1 (n=1)  Collaborative Cross (n=1) | | Both (n=1) | | 1 (n=1) | | 2–7 days (n =1) | |
| **The following systems were listed in alphabetical order.** | | | | | | | | | | | | | |  |
| 24 channel activity monitoring system (O'Hara & Co., Tokyo, Japan) | | | 3 | | Mouse (n=3) | | C57BL/6 (n=3) | | Male (n=3) | | 1 (n=2)  4 (n=1) | | 2–7 days (n=1)  8–14 days (1–2 weeks) (n=1)  29–84 days (1–3 months) (n=1) |  |
| ACTIMO System (Shintechno, Japan) | | | 1 | | Rat (n=1) | | Wistar (n=1) | | Male (n=1) | | 1 (n=1) | | 8–14 days (1–2 weeks) (n=1) |  |
| ActiviScope (NewBehavior, Switzerland) | | | 3 | | Mouse (n=2)  Rat (n=1) | | C57BL/6 (n=1)  Mixed background (n=1)  Wistar (n=1) | | Male (n=2)  N/A (n=1) | | 1 (n=3) | | 15–28 days (2–4 weeks) (n=1)  29–84 days (1–3 months) (n=1)  N/A (n=1) |  |
| Activity cages, UMOTWin (Ellegaard systems, Denmark) | | | 1 | | Mouse (n=1) | | Mixed background (n=1) | | Both (n=1) | | 1 (n=1) | | 2–7 days (n=1) |  |
| Activity monitor (AccuScan Instruments, USA) | | | 4 | | Mouse (n=4) | | C57BL/6 (n=3)  129 (n=1)  WSC (n=1)  WSP (n=1) | | Male (n=3)  Female (n=1) | | 1 (n=4) | | 8–14 days (1–2 weeks) (n=2)  15–28 days (2–4 weeks) (n=1)  29–84 days (1–3 months) (n=1) |  |
| Activity sensor (Neuroscience Inc., Japan) | | | 1 | | Mouse (n=1) | | C57BL/6 (n=1)  BALB/c (n=1) | | Male (n=1) | | N/A (n=1) | | 29–84 days (1–3 months) (n=1) |  |
| Activity sensor and food intake monitor (O'Hara & Co., Tokyo, Japan) | | | 1 | | Rat (n=1) | | Wistar (n=1) | | Male (n=1) | | 1 (n=1) | | 29–84 days (1–3 months) (n=1) |  |
| Animex activity meter (LKB Instruments, Sweden) | | | 2 | | Mouse (n=1)  Rat (n=1) | | C57BL/6 (n=1)  Sprague Dawley (n=1) | | Male (n=2) | | 2 (n=1)  10 (n=1) | | 15–28 days (2–4 weeks) (n=1)  29–84 days (1–3 months) (n=1) |  |
| Any-maze Cage (Stoelting, Ireland) | | | 1 | | Mouse (n=1) | | C57BL/6 (n=1) | | Male (n=1) | | 1 (n=1) | | 8–14 days (1–2 weeks) (n=1) |  |
| Automated drinking & feeding monitor system (TSE, USA/Germany) | | | 1 | | Mouse (n=1) | | C57BL/6 (n=1)  NZB (n=1)  NZO (n=1) | | Male (n=1) | | n (n=1) | | N/A (n=1) |  |
| BASi (West Lafayette, USA) | | | 1 | | Rat (n=1) | | Wistar (n=1) | | N/A (n=1) | | N/A (n=1) | | 8–14 days (1–2 weeks) (n=1) |  |
| BioDAQ food intake monitoring system (Research Diets, USA) | | | 1 | | Rat (n=1) | | Wistar (n=1) | | Female (n=1) | | 1 (n=1) | | N/A (n=1) |  |
| CI Multi-Device Interface Multi Device Interface MDI Software (Columbus Instruments, USA) | | | 1 | | Mouse (n=1) | | C57BL/6 (n=1) | | Male (n=1) | | 1 (n=1) | | 15–28 days (2–4 weeks) (n=1) |  |
| Comprehensive Laboratory Animal Monitoring System (CLAMS, Columbus Instruments, USA | | | 1 | | Mouse (n=1) | | C57BL/6 (n=1) | | Both (n=1) | | 1 (n=1) | | 169–336 days (6–12 months) (n=1) |  |
| Digiscan (AccuScan Instruments, USA) | | | 1 | | Mouse (n=1) | | BALB/c (n=1)  C57BL/6 (n=1)  DBA/2 (n=1) | | Male (n=1) | | 1 (n=1) | | 29–84 days (1–3 months) (n=1) |  |
| Digital scale (EAGDCE-L, Sartorius AG, Germany) | | | 1 | | Rat (n=1) | | Sprague Dawley (n=1) | | Male (n=1) | | 2 (n=1) | | 15–28 days (2–4 weeks) (n=1) |  |
| Doppler radar (Model BBL; McEwan Technologies, USA) | | | 1 | | Rat (n=1) | | Wistar (n=1) | | Male (n=1) | | 1 (n=1) | | N/A (n=1) |  |
| Infrared non-contact thermal imager (Flir Systems, ThermoVision A320, USA) | | | 1 | | Rat (n=1) | | Wistar (n=1) | | Male (n=1) | | 1 (n=1) | | N/A (n=1) |  |
| Model 12 polygraph (Grass-Telefactor, USA) | | | 1 | | Rat (n=1) | | Wistar (n=1) | | Male (n=1) | | 1 (n=1) | | N/A (n=1) |  |
| Drinkometer system, MOUSE-E-MOTION (INFRA-E-MOTION GmbH, Germany) | | | 1 | | Mouse (n=1) | | C57BL/6 (n=1) | | Male (n=1) | | 1 (n=1) | | 29–84 days (1–3 months) (n=1) |  |
| DVC (Tecniplast, Italy) | | | 5 | | Mouse (n=5) | | C57BL/6 (n=4)  BALB/c (n=1) | | Male (n=1)  Both (n=3)  N/A (n=1) | | 1 (n=1)  2 (n=1)  3 (n=1)  4 (n=1)  5 (n=1) | | 2–7 days (n=1)  29–84 days (1–3 months) (n=1)  85–168 days (3–6 months) (n=2)  337 and more days (> 1 year) (n=1) |  |

| EthoVision (Noldus, the Netherlands) | 4 | Mouse (n=4) | C57BL/6 (n=3)  Mixed background (n=1) | Male (n=2)  Both (n=1)  N/A (n=1) | 1 (n=3)  N/A (n=1) | 8–14 days (1–2 weeks) (n=1)  N/A (n=3) |
| --- | --- | --- | --- | --- | --- | --- |
| Feeding chambers (Med Associates, USA) | 1 | Rat (n=1) | Sprague Dawley (n=1)  Wistar (n=1) | Male (n=1) | 2 (n=1) | 29–84 days (1–3 months) (n=1) |
| HCA (Actual analytics, United Kingdom) | 5 | Mouse (n=2)  Rat (n=3) | C57BL/6 (n=2)  C3H (n=1)  Sprague Dawley (n=1)  Wistar (n=2) | Male (n=1)  Both (n=3)  N/A (n=1) | 1 (n=1)  2 (n=1)  3 (n=1)  4 (n=1)  5 (n=1) | 2–7 days (n=1)  29–84 days (1–3 months) (n=1)  85–168 days (3–6 months) (n=2)  337 and more days (> 1 year) (n=1) |
| HM-2, MBRose, Denmark | 1 | Mouse (n=1) | C57BL/6 (n=1) | Male (n =1) | 4 (n=1) | 29–84 days (1–3 months) (n=1) |
| Home Cage Activity System (Coulbourn Instruments, Allentown, PA, USA) | 3 | Mouse (n=2)  Rat (n=1) | C57BL/6 (n=2)  Wistar (n=1) | Male (n=3) | 1 (n=3) | 2–7 days (n=2)  15–28 days (2–4 weeks) (n=1) |
| HomeCageScan (Cleversys, USA) | 10 | Mouse (n=8)  Rat ( n=2) | C57BL/6 (n=1)  CBA (n=1)  DBA/2 (n=1)  NOD (n=1)  Swiss Webster (n=1)  129 (n=1)  C57BL/6 (n=3)  Mixed background n=3)  Long Evans (n=1)  Sprague Dawley (n=2) | Male (n =5)  Female (n=1)  Both (n=2)  N/A (n=2) | 1 (n=10) | 2–7 days (n=3)  8–14 days (1–2 weeks) (n=1)  15–28 days (2–4 weeks) (n=1)  29–84 days (1–3 months) (n =4)  N/A (n=1) |
| iButtons (Maxim Integrated Products, United Kingdom) | 1 | Mouse (n=1) | C57BL/6 (n=1)  129 (n=1)  Mixed background (n=1) | Male (n=1) | 1 (n=1) | 29–84 days (1–3 months) (n =1) |
| InfraMot (TSE, USA/Germany) | 6 | Mouse (n=4)  Rat (n=2) | C57BL/6 (n=4)  Sprague Dawley (n=2)  Wistar (n=1) | Male (n=1)  Female (n=1)  Both (n=3)  N/A (n=1) | 1 (n=5)  5 (n=1) | 1 day (n=1)  2–7 days (n=2)  8–14 days (1–2 weeks) (n=1)  15–28 days (2–4 weeks) (n=1)  29–84 days (1–3 months) (n =1) |
| Infrared Motion Detector (Starr Life Sciences, USA) | 2 | Rat (n=2) | Sprague Dawley (n=2) | Male (n=1)  Both (n=1) | 1 (n=1)  6 (n=1) | 8–14 days (1–2 weeks) (n=1)  85–168 days (3–6 months) (n=1) |
| Infrared motion sensors (ClockLab, Actimetrics, USA) | 1 | Mouse (n=1) | Mixed background (n=1) | Male (n=1) | 1 ( n=1) | 29–84 days (1–3 months) (n =1) |
| Intellicage (TSE, USA/Germany) | 9 | Mouse (n=8)  Rat (n=1) | C57BL/6 (n=5)  BALB/c (n=3)  DBA/2 (n=3)  129 (n=1)  Mixed background (n=3)  Sprague Dawley (n=1) | Male (n=2)  Female (n=4)  Both (n=3) | 4 (n=1)  7 (n=1)  8 (n =2)  10 (n=3)  > 10 (n=2) | 2–7 days (n=1)  8–14 days (1–2 weeks) (n=2)  15–28 days (2–4 weeks) (n=3)  29–84 days (1–3 months) (n =1)  169–336 days (6–12 months) (n=1)  N/A (n=1) |
| LabMaster, Environmental chamber, WB 2000 KHL (TSE, USA/Germany) | 1 | Mouse (n=1) | C57BL/6 (n=1) | Male (n=1) | N/A (n=1) | 15–28 days (2–4 weeks) (n=1) |
| Laboras (Metris, the Netherlands) | 4 | Mouse (n=3)  Rat (n=1) | C57BL/6 (n=3)  Sprague Dawley (n=1) | Male (n=3)  Female (n=1) | 1 (n=3)  2 (n=1) | 15–28 days (2–4 weeks) (n=1)  29–84 days (1–3 months) (n =2)  N/A (n=1) |
| Marlau cage (ViewPoint Behavior Technology) | 1 | Rat (n=1) | Sprague Dawley (n=1) | Male (n=1) | > 10 (n=1) | 85–168 days (3–6 months) (n=1) |
| MicroMax Activity monitor (AccuScan Instruments, USA) | 2 | Mouse (n=2) | C57BL/6 (n=1)  CD-1 (n=1) | Male (n=2) | 1 (n=1)  4 (n=1) | 1 day (n=1)  8–14 days (1–2 weeks) (n=1) |
| Mini Run-a-Round (Pets International, Elk Grove Village, IL, USA) | 1 | Mouse (n=1) | C57BL/6 (n=1)  129 (n=1) | Both (n=1) | 1 (n=1) | 85–168 days (3–6 months) (n=1) |
| Nano tag (ACOS, Nagano, Japan) | 1 | Rat (n=1) | Fischer 344 (n=1) | Male (n=1) | 3 (n=1) | 29–84 days (1–3 months) (n =1) |
| Noninvasive sleep-monitoring apparatus (Signal Solutions, Lexington, KY) | 1 | Mouse (n=1) | BALB/c (n=1)  C57BL/6 (n=1)  CD-1 (n=1) | Both (n=1) | 1 (n=1) | 15–28 days (2–4 weeks) (n=1) |
| Operant chambers (Coulbourn Instruments, USA) | 2 | Rat (n=2) | Sprague Dawley (n=2) | Male (n=2) | 1 (n=2) | 8–14 days (1–2 weeks) (n=1)  15–28 days (2–4 weeks) (n=1) |
| Operant chambers (MED Associates, USA) | 3 | Mouse (n=1)  Rat (n=2) | C57BL/6 (n=1)  DBA/2 (n=1)  Long Evans (n=1)  MR (n=1)  MNRA (n=1) | Male (n=3) | 1 (n=3) | 8–14 days (1–2 weeks) (n=2)  N/A (n=1) |
| Opto-M3 Dual Axis System (Columbus Instruments, USA) | 4 | Mouse (n=1)  Rat (n=3) | C57BL/6 (n=1)  Lister hooded (n=2)  Sprague Dawley (n=1) | Male (n=3)  Both (n=1) | 1 (n=4) | 2–7 days (n=2)  15–28 days (2–4 weeks) (n=1)  N/A (n=1) |
| PhenoMaster (TSE, USA/Germany) | 7 | Mouse (n=5)  Rat (n=2) | C57BL/6 (n=4)  DBA/2 (n=1)  129 (n=1)  C3H (n=1)  Mixed background (n=2)  Long Evans (n=1)  Sprague Dawley (n=2) | Male (n=3)  Female (n=1)  Both (n=3) | 1 (n=6)  10 (n=1) | 2–7 days (n=3)  15–28 days (2–4 weeks) (n=1)  29–84 days (1–3 months) (n =1)  85–168 days (3–6 months) (n=1)  169–336 days (6–12 months) (n=1) |
| Phenotyper (Noldus, the Netherlands) | 26 | Mouse (n=23)  Rat (n=3) | C57BL/6 (n=21)  DBA/2 (n=8)  129 (n=4)  A/J (n=3)  BTBR (n=2)  FVB (n=2)  AKR (n=1)  BALB/c (n=2)  C3H (n=2)  NOD (n=2)  WSB (n=1)  PWK (n=1)  CAST (n=1)  Mixed background (n=2)  Sprague Dawley (n=2)  Wistar (n=1) | Male (n=15)  Female (n=2)  Both (n=8)  N/A (n=1) | 1 (n=16)  2 (n=2)  3 (n=1)  4 (n=3)  5 (n=1)  10 (n=1)  N/A (n=2) | 2–7 days (n=13)  8–14 days (1–2 weeks) (n=4)  15–28 days (2–4 weeks) (n=6)  29–84 days (1–3 months) (n =3) |
| PhenoWorld (TSE, USA/Germany) | 1 | Rat (n=1) | Wistar (n=1) | Male (n=1) | 6 (n=1) | 29–84 days (1–3 months) (n =1) |
| Photobeam Activity System (Columbus Instruments, USA) | 1 | Mouse (n=1) | C57BL/6 (n=1) | Male (n=1) | 1 (n=1) | N/A (n=1) |
| Photobeam Activity System-Home Cage (San Diego Instruments) | 3 | Mouse (n=3) | C57BL/6 (n=2)  Mixed background (n=1)  129 (n=1) | Both (n=1)  Male (n=2) | 1 (n=3) | N/A (n=1)  8–14 days (1–2 weeks) (n=1)  29–84 days (1–3 months) (n =1) |
| Physiobelt system (Open Science, Russia) | 1 | Rat (n=1) | Long Evans (n=1) | Male (n=1) | 1 (n=1) | 2–7 days (n=1) |
| Radiotelemeter catheter (DSI, USA) | 1 | Mouse (n=1) | C57BL/6 (n=1) | Male (n=1( | N/A (n=1) | 2–7 days (n=1) |
| SAMAB (System for Automated Measurement of Behaviour) | 1 | Mouse (n=1) | C57BL/6 (n=1) | Male (n=1) | 1 (n=1) | 29–84 days (1–3 months) (n =1) |
| Selective Activity Meter (Model S) (Columbus Instruments, USA) | 1 | Mouse (n=1) | OF-1 (n=1) | Male (n=1) | 6 (n=1) | 15–28 days (2–4 weeks) (n=1) |
| SmartFrame (Lafayette Instrument, USA) | 1 | Mouse (n=1) | FVB (n=1) | Male (n=1) | 1 (n=1) | 29–84 days (1–3 months) (n =1) |
| TopScan (CleverSys, USA) | 1 | Mouse (n=1) | C57BL/6 (n=1) | Male (n=1) | 1 (n=1) | 8–14 days (1–2 weeks) (n=1) |
| Videotrack (O'Hara & Co., Tokyo, Japan) | 1 | Mouse (n=1) | C57BL/6 (n=1) | Male (n=1) | 2 (n=1) | 2–7 days (n=1) |
| Vium Digital Smart Houses (San Mateo, CA , USA) | 1 | Rat (n=1) | Lewis (n=1) | Male (n=1) | 2 (n=1) | 15–28 days (2–4 weeks) (n=1) |
| Winchester (5 led), EEG recording system (2 Grass, 12 tracks, 79D model), rotating collector (APCL 12 channels, Air precision) | 1 | Mouse (n=1) | Swiss Webster (n=1) | Male (n=1) | 1 (n=1) | 15–28 days (2–4 weeks) (n=1) |
