# Supplementary material for: A systematic review of the development and application of home cage monitoring in laboratory mice and rats
Source: BMC Biol. 2023 Nov 13;21:256. doi: 10.1186/s12915-023-01751-7 (PMC10642068; doi:10.1186/s12915-023-01751-7)
Supplement: Supplementary file 4 — Additional file 4. Social housing structure in single-sex studies (data obtained from n = 187 single-sex studies including mice and n = 191 single-sex studies including rats). [file 12915_2023_1751_MOESM4_ESM.docx]

**Percentage of single-sex studies in which males and females were housed individually or in groups.**

|  | **Individual housing**  (%, abs. number given in brackets) | **Group housing**  (%, abs. number given in brackets) | **Not indicated**  (%, abs. number given in brackets) |
| --- | --- | --- | --- |
| **Mice** |  |  |  |
| Males | 68 (102) | 26 (39) | 7 (10) |
| Females | 36 (13) | 61 (22) | 3 (1) |
| **Rats** |  |  |  |
| Males | 62 (103) | 33 (55) | 4 (7) |
| Females | 65 (17) | 23 (6) | 12 (3) |
| Legend: For this table, publications in which both species or both sexes of a species were used or the sex of the animals was not indicated were excluded. Due to rounding, the numbers do not always sum to 100% | | | |

Considering housing conditions for single-sex studies, 68% of studies including male mice only used individual housing, while only 36% of the studies including female mice only kept the animals in social isolation. In contrast to studies including male mice only, most studies using exclusively female mice (61%) kept them in groups. For rats, both sexes (62% of the males and 65% of the females) were predominantly single-housed.

These data must be interpreted with caution because of the huge bias towards male-only studies included in the present systematic review (Table 1). Moreover, single-sex studies including males or females only may involve different procedures requiring individual or social housing.
